# Serum concentrations of amino acids and tryptophan metabolites are affected by consumption of a light breakfast: a clinical intervention study in adults with overweight or obesity

**DOI:** 10.1186/s40795-022-00661-1

**Published:** 2023-01-11

**Authors:** Ingrid V. Hagen, Anita Helland, Marianne Bratlie, Øivind Midttun, Adrian McCann, Arve Ulvik, Gunnar Mellgren, Per M. Ueland, Oddrun A. Gudbrandsen

**Affiliations:** 1 Dietary Protein Research Group, Department of Clinical Medicine, University of Bergen, 5021 Bergen, Norway; 2 Bevital AS, Jonas Lies Veg 87, 5021 Bergen, Norway; 3 Mohn Nutrition Research Laboratory, Department of Clinical Science, University of Bergen, Haukeland University Hospital, 5021 Bergen, Norway; 4 Hormone Laboratory, Department of Medical Biochemistry and Pharmacology, Haukeland University Hospital, 5021 Bergen, Norway

**Keywords:** Kynurenines, Amino acids

## Abstract

**Background:**

Epidemiological studies often investigate amino acids and their metabolites as biomarkers, but do not always consistently use fasting or non-fasting blood samples, or may lack information on the prandial status of the study participants. Since little information is available on the effects of the prandial status on many biomarkers, and since blood is typically sampled early in the day with participants in a fasting state or after having consumed a light meal in many trials, the main purpose of this study was to investigate the short-term effects of a light breakfast on serum concentrations of amino acids and related metabolites.

**Methods:**

Blood was collected from sixty-three healthy adults (36 women) in the fasting state and at set times for 120 min after intake of a light breakfast with low protein content (14 g protein, 2218 kJ). Relative changes in serum biomarker concentrations from fasting to postprandial serum concentrations were tested using T test.

**Results:**

The serum concentrations of 13 of the 20 measured amino acids were significantly changed 60 min following breakfast intake, with the most marked effects seen as increases in alanine (34%) and proline (45%) concentrations. The response did not reflect the amino acid composition of the breakfast. The concentrations of seven kynurenine metabolites were significantly decreased after breakfast.

**Conclusion:**

Consumption of a light breakfast affected serum concentrations of several amino acids and related metabolites, underlining the importance of having information regarding the participants’ prandial state at the time of blood sampling in studies including these biomarkers.

**Trial registration:**

This trial was registered at clinicaltrials.gov as NCT02350595 (registered January 2015).

## Background

Inconsistent observations in epidemiological studies investigating biomarkers in relation to various health and disease outcomes may be linked to differences in blood collection protocols. Especially, it is unclear how the time interval between last meal and blood draw may affect biomarker concentrations in blood. In the fasting state, circulating concentrations of glucose and insulin decline after last food intake, and free amino acids secreted from muscles and the gut are mainly directed to the liver for gluconeogenesis or ketogenesis. Glucose, produced by the liver, is taken up by muscle cells and oxidized to pyruvate which undergoes transamination to form alanine. Alanine returns to the liver where it’s carbon skeleton enters gluconeogenesis thus completing the glucose-alanine cycle [1]. In muscle, branched chain amino acids (BCAA) are the main source used for alanine synthesis, as BCAAs are catabolised through transamination to form glutamate, which together with pyruvate forms alanine and α-ketoglutarate in a second transamination reaction [2]. Although other amino acids are also produced from pyruvate in muscle, alanine and glutamine are the main amino acids that leave the muscle [2], and the hepatic capacity for gluconeogenesis is larger from alanine compared to all other amino acids [1].

In the postprandial state, the increased insulin and lower glucagon concentrations stimulate amino acid uptake in muscle, especially of BCAAs, for protein synthesis, and thus inhibiting proteolysis. However, insulin seems to not inhibit release of alanine from muscle [1], and in this phase, the gluconeogenesis is inhibited because insulin reduces the uptake of alanine and other glucogenic substrates by the liver, and the release of glucose from liver is reduced [1]. The suppression of gluconeogenesis is compromised in patients with impaired glucose tolerance [3].

In patients with insulin resistance, the muscle protein breakdown is less inhibited by insulin [4], and elevated fasting and postprandial concentrations of BCAAs have been found in adults with obesity when compared to normal weight adults [5–8]. In addition, high fasting concentrations of BCAAs and the amino acid catabolites α-hydroxybutyrate (from threonine and methionine), β-hydroxyisobutyrate (from valine) and α-ketoglutarate (produced from glutamate by transamination) are associated with impaired glucose regulation [6, 9–11].

The degradation of the essential amino acid tryptophan is well characterized and has been studied in relation to several metabolic disturbances as well as a plethora of cancerous, inflammatory and other diseases [12]. Tryptophan is mainly catabolised through the kynurenine pathway [13], which is controlled at its first step, catalysed by the rate-determining enzymes tryptophan 2,3-dioxygenase in liver and indoleamine 2,3-dioxygenase in extrahepatic tissues [14]. It is of interest that the tryptophan 2,3-dioxygenase activity in liver is inhibited by glucose intake in rats, probably involving increased production of the feedback allosteric inhibitor NADPH [15]. This may explain the associations between elevated concentrations of kynurenine metabolites with impaired glucose tolerance observed in individuals with overweight or obesity [16, 17]. Thus, the possible inhibitory effect of glucose on the kynurenine pathway underscores the importance of investigating if the prandial status can impact the concentrations of the metabolites in this pathway.

Epidemiological studies do not always consistently use fasting or non-fasting blood samples, or may lack information of the prandial status of the study participants, and in many trials blood is typically sampled early in the day during working hours for practical reasons, i.e. with participants in a fasting state or after having consumed a light meal. Therefore, the main aim of this study was to evaluate the effects of a light breakfast on serum concentrations of amino acids, kynurenine pathway metabolites, and a selection of metabolites related to glucose regulation. The secondary aim of this study was to compare all measured biomarkers between men and women. Blood samples were collected in fasting state and at set times for 120 min after intake of a breakfast with low protein content (14 g) but relatively high carbohydrate content (80 g), with the intention to investigate the importance of having fasting samples for measurements of these biomarkers. Glucose and insulin were quantified in fasting and postprandial serum samples to verify the responses after food intake. Our hypothesis was that when the participants’ metabolic status changed from catabolic to anabolic after intake of a light meal, this would affect concentrations of both amino acids and relevant metabolites in serum.

## Methods

### Participants, study setting and ethics

The subjects in the present work were participants in a study that was designed to investigate the effects of high intake of fish (5 dinners per week) for 8 weeks. In the present paper we present analysis of the samples collected at baseline. The study design, as well as description of study participants, study setting and protocol for study visits, have previously been described in detail [18]. In brief, the study population consisted of adults of Norwegian ethnic origin (Caucasian) with overweight or obesity living in Bergen, Norway. Inclusion criteria were BMI ≥ 27 kg/m2, fasting blood glucose ≤ 7.0 mmol/l, and age 18–69 years. Exclusion criteria were pregnancy, incompatibility with fish consumption (allergies, intolerance and/or dislike), diagnosed diabetes mellitus, heart disease or gastrointestinal disease, use of medications affecting lipid metabolism or glucose homoeostasis, use of anti-inflammatory medications, use of supplements containing n-3 polyunsaturated fatty acids (PUFA), intentional weight loss and large fluctuation in body weight (> 3 kg) during the preceding 2 months. Seventy-six participants were included [19], and 68 participants completed the trial. Three participants were excluded (one had prediabetes and two participants did not comply with the protocol). For two participants, we did not have a sufficient amount of blood serum for analyses; hence, the present dataset consists of serum from 63 participants. All participants had serum creatinine concentration and urine albumin:creatinine ratio within normal ranges [20]. Examinations were conducted at the Clinical Research Unit at the Haukeland University Hospital, Bergen, Norway.

The study was conducted according to the guidelines laid down in the Declaration of Helsinki, and all procedures were approved by the Regional Committee for Medical and Health Research Ethics of Western Norway (REC no.: 2011/572). Written informed consent was obtained from all participants.

Health professionals performing blood sampling, and personnel conducting the laboratory analyses, were all blinded to the participants’ identity, and all data were analysed anonymously. The trial is registered at clinicaltrials.gov as NCT02350595 (29/01/2015).

### Protocol for study visits

Examinations and samplings were conducted in the morning after an overnight fast. The participants were instructed not to eat or drink anything except water, or use substances containing nicotine after 10 pm the previous day, and to avoid physical exercise and alcohol for 24 h before the visit. Body weight and body composition were measured using a bioelectrical impedance analysis device (InBody 720; Biospace Co. Ltd) in a fasted state. Participants completed dietary records of the 5 preceding days before the visit, including at least one weekend-day, and daily protein intake was calculated using the ‘Mat på Data 5.1’ software [21] and information provided by the manufacturers. Blood was collected by venepuncture by inserting a cannula connected to a three-way tap for repeated measures and the system was flushed with sterile saline (0.9%) before and after each blood sample. Blood was collected in BD Vacutainer SST II Advance gel tubes (Becton, Dickinson and Company) for isolation of serum. The staff complied with a strict protocol for pre-analytical sample handling to ensure high sample quality. Blood samples were centrifuged after 30 min at room temperature, and serum samples were immediately aliquoted and frozen at − 80 °C until analyses.

### Intervention

After the collection of fasting blood samples, the participants ingested a standardized breakfast consisting of one slice of white bread with 5 g margarine and 25 g strawberry jam, one slice of white bread with 5 g margarine and 20 g white cheese, and 0.30 L orange juice. The estimated contents of macronutrient and energy in the standardised breakfast were calculated using 'Mat på Data 5.1' [21] and information provided by the manufacturers, and an overview of the macronutrients in the breakfast is presented in Table 1. The protein content in food in databases is based on total nitrogen content determined using the Kjeldahl method with a nitrogen factor of 6.25, and is a rough estimation that includes organic nitrogen containing compounds and ammonium/ammonia. The analysis of amino acids in the breakfast was conducted by Nofima BioLab (Bergen, Norway) using the methods by Cohen et al. [22] and Miller et al. [23]. The breakfast was consumed within 15 min. Blood samples were collected in fasting state and 30, 60, 90 and 120 min after the participants had consumed the standardized breakfast. Serum from fasting state and after 60 and 120 min following breakfast was sampled for analyses of amino acids and relevant metabolites including metabolites in the kynurenine pathway, serum collected in fasting state and 30, 60, 90 and 120 min following breakfast intake were sampled for analyses of glucose, and serum from fasting state and 120 min postprandially were sampled for analyses of insulin.

**Table 1:** Estimated contents of energy and macronutrients in the standardized breakfast^1^

	Energy, kJ	Carbohydrates, g	Protein, g	Fat, g
White bread^2^, 75 g	820	35	7	2
Margarine, 10 g	297	0	0	8
Strawberry jam, 25 g	261	15	0	0
White cheese, 20 g	295	0	5	5
Orange juice, 0.30 L	545	30	2	1
Total	2218	80	14	16

^1^Calculated using 'Mat på Data 5.1' [21] and information provided by the manufacturers

^2^Two slices

### Analyses in serum

Serum concentrations of amino acids, metabolites in the kynurenine pathway, and the amino acid catabolites α-hydroxybutyrate, β-hydroxyisobutyrate and α-ketoglutarate were measured by Bevital AS (Bergen, Norway, http://www.bevital.no). In brief, amino acids (except arginine), α-hydroxybutyrate, β-hydroxyisobutyrate and α-ketoglutarate were measured by mixing 50 µL of serum with dithioerythritol, ethanol, and isooctane/chloroform, and centrifuged. The organic layer was removed, and the aqueous fraction was mixed with ethanol, water, pyridine, and methylchloroformate (in toluene) to derivatise the water-soluble biomarkers (making them collect in the organic toluene phase) before analysis by GC–MS/MS using a CP Sil 24-CB low-bleed/MS capillary column from Varian [24]. Arginine was quantified by mixing 45µL of serum with trichloroacetic acid, followed by centrifugation, and injecting the supernatant onto a Fortis Phenyl column with a mobile phase consisting of water, acetic acid and methanol for analysis by HPLC–MS/MS [25]. For analysis of tryptophan metabolites, proteins were precipitated from 60 µL of serum by addition of trichloroacetic acid. After centrifugation, the supernatant was injected onto a Zorbax stable-bond C8 reversed-phase column system using a gradient mobile phase containing acetic acid, heptafluorobutyric acid and acetonitrile for analysis by HPLC–MS/MS [26]. Matching isotope-labeled internal standards were used for all analytes. Serum glucose and insulin were analysed by routine methods at the Department of Medical Biochemistry and Pharmacology, Haukeland University Hospital (Bergen, Norway). Glucose was analysed on a Modular P instrument (Roche Diagnostics GmbH), using the Gluco-quant Glucose/HK enzymatic assay (Roche Diagnostics). Insulin was analysed on the Immulite 2000 Immunoassay System (Siemens Healthcare GmbH), using the IML.2000 Insulin kit 600 T (Siemens). All serum samples for each analysis were analysed for each participant in random order on the same day, and samples were not thawed previously. The serum samples had low or no haemolysis (HIL index < 0.5 g/L).

### Outcome measurements

The primary outcome of the present study was to compare serum concentrations of amino acids, the kynurenine pathway metabolites, and a selection of amino acid catabolites related to glucose regulation in fasting serum and serum collected 60 and 120 min after intake of a standardised breakfast. The secondary outcome was to compare the measured serum biomarker concentrations and relative changes in these from fasting to postprandial between men and women.

### Sample size estimation

The present study exploits biological material, anthropometric data and information collected at the baseline visit in an intervention study that was designed to investigate the effects of high intake of cod or salmon on post-prandial glucose regulation after a standardised breakfast in participants with overweight or obesity [18]. The sample size estimation for the original study showed that it was necessary to include seventy-six participants divided into three groups to ensure that twenty participants in each group completed the trial with satisfactory compliance, with a power of 80% and α of 0.05 [18]. Since the present study is, to the best of our knowledge, the first study to investigate the effects of a light breakfast on a panel of amino acids and metabolites in healthy adults with overweight or obesity, data on effect size were not available for sample size calculation or minimally detectable effect sizes for the present study.

### Statistical analyses

The serum concentrations of amino acids and metabolites including kynurenines were generally not normally distributed, and the fasting concentrations are therefore presented as geometric means with 95% confidence intervals. For postprandial samples, the analyte ratios relative to fasting concentration were used for testing in order to make the resulting ratios comparable across biomarkers. One-sided T test was used to test if the ratios at each follow-up were different from 1 (fasting), and paired T test was used to test if the ratios in the postprandial samples were different from each other. Genders were compared using Welch's T test for independent samples. All results from t-tests were Benjamini–Hochberg adjusted, and *p* < 0.05 were considered statistically significant. All statistical tests were performed using R version 4.0.3 (http://www.r-project.org).

## Results

### Participant characteristics

Sixty-three participants (36 women) were included in the present study, with a geometric mean (5, 95% CI) age of 42.8 (39.9, 45.8) years and geometric mean BMI (5, 95% CI) of 32.9 (31.8, 34.0) kg/m^2^ (Table 2). The geometric means of age, BMI, body weight and the fasting serum concentrations of glucose and insulin were similar between the genders, whereas the percent muscle mass was significantly higher in men and the percent total body fat was significantly higher in women. The geometric mean for estimated daily protein intake for the 5 days preceding the visit was significantly higher in men compared to women (Table 2).

**Table 2:** Age, anthropometry, fasting serum concentrations of glucose and insulin, and estimated daily protein intake

	Total (*N* = 63)	Men (*N* = 27)	Women (*N* = 36)	*P* sex
Age (years)	42.8 (39.9, 45.8)	44.3 (40.6, 48.2)	41.6 (37.4, 46.3)	1.00
BMI (kg/m^2^)	32.9 (31.8, 34.0)	32.9 (31.3, 34.6)	32.8 (31.3, 34.4)	1.00
Body weight (kg)	98.3 (94.3, 102.6)	106.0 (99.2, 113.2)	92.6 (88.3, 97.2)	0.088
Muscle mass (%)	34.6 (33.4, 35.8)	38.8 (37.2, 40.5)	31.5 (30.6, 32.4)	1.4 × 10^–8^
Body fat (%)	36.9 (34.8, 39.1)	30.7 (28.3, 33.4)	42.7 (41.2, 44.2)	8.5 × 10^–7^
Glucose (mmol/l)	5.2 (5.1, 5.4)	5.3 (5.1, 5.5)	5.2 (5.0, 5.4)	1.00
Insulin (pmol/l)	7.5 (6.2, 9.0)	8.4 (6.4, 11.1)	6.7 (5.2, 8.6)	1.00
Protein intake (g/day)^1^	92 (87, 98)	106 (97, 116)	83 (77, 89)	0.0039

^1^Geometric mean of estimated daily protein intake for 5 days preceding the blood sampling

Data are presented as geometric means with 5, 95% CI. Men and women were compared using independent samples T test. *P* < 0.05 was considered significant

### Description of the standardised breakfast

Analyses of amino acid composition in the breakfast showed the highest content of glutamate + glutamine (3.06 g), with proline as the second highest (1.84 g), and between 0.5 and 1 g each of isoleucine, leucine, lysine, phenylalanine, valine, arginine, aspartate + asparagine and serine, and below 0.5 g of alanine (Table 3). Contents of histidine, methionine, threonine, tryptophan, cysteine + cystine, glycine and tyrosine were below detection levels (0.10 g/100 g sample, corresponding to 0.44 g per breakfast serving). The sum of quantified amino acids in the breakfast amounted to 11.11 g, which is somewhat lower than the estimated protein content of 14 g in the breakfast based on information in the Norwegian database 'Mat på Data 5.1' [21] and the information provided by the manufacturers (Table 1).

**Table 3:** Amino acid content in the standardized breakfast

	g/breakfast serving
Essential amino acids	
Histidine	< LOD
Isoleucine	0.52
Leucine	1.01
Lysine	0.61
Methionine	< LOD
Phenylalanine	0.66
Threonine	< LOD
Tryptophan	< LOD
Valine	0.66
Non-essential amino acids	
Alanine	0.44
Arginine	0.74
Aspartate + Asparagine	0.92
Cysteine + Cystine	< LOD
Glutamate + Glutamine	3.06
Glycine	< LOD
Proline	1.84
Serine	0.66
Tyrosine	< LOD

Means of two measurements; deviations were < 5% between parallels. Level of detection (LOD) for amino acids is 0.10 g/100 g sample, corresponding to 0.44 g per breakfast serving

### Effects of breakfast intake on serum amino acids and selected metabolites

**Fig. 1:**
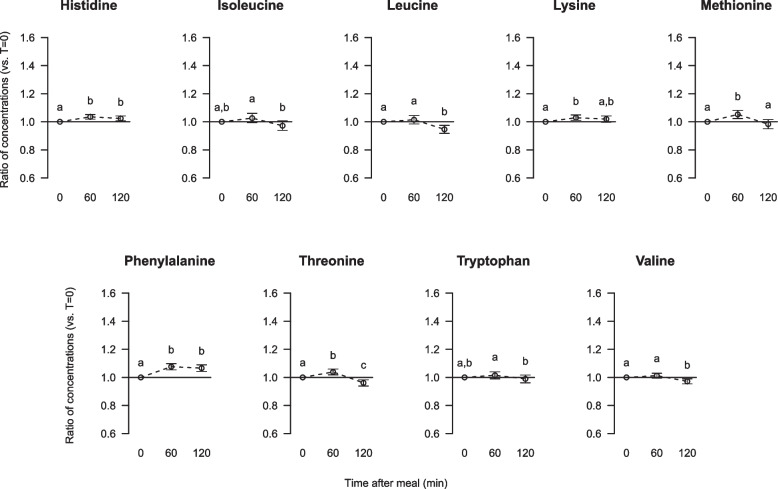
Relative changes from fasting to postprandial serum concentrations of essential amino acids. Data are presented as ratios with 5, 95% CI for 63 participants. Different letters (**a**, **b**, **c**) indicate significant differences between the time points 0 (fasting), 60 and 120 min; *P* < 0.05 was considered significant

The serum concentrations of the essential amino acids (EAA) histidine and phenylalanine were significantly increased (Fig. 1). The concentrations of leucine and valine were not changed after 60 min but were reduced 120 min after consumption of the breakfast. Threonine and methionine concentrations were initially significantly increased after 60 min and thereafter significantly decreased, and lysine concentration was significantly increased after 60 min and was similar to fasting concentration after 120 min. All the statistically significant changes from fasting to postprandial concentration were < 10% for these EAA. Serum concentrations of isoleucine and tryptophan were not affected by the meal.

**Fig. 2:**
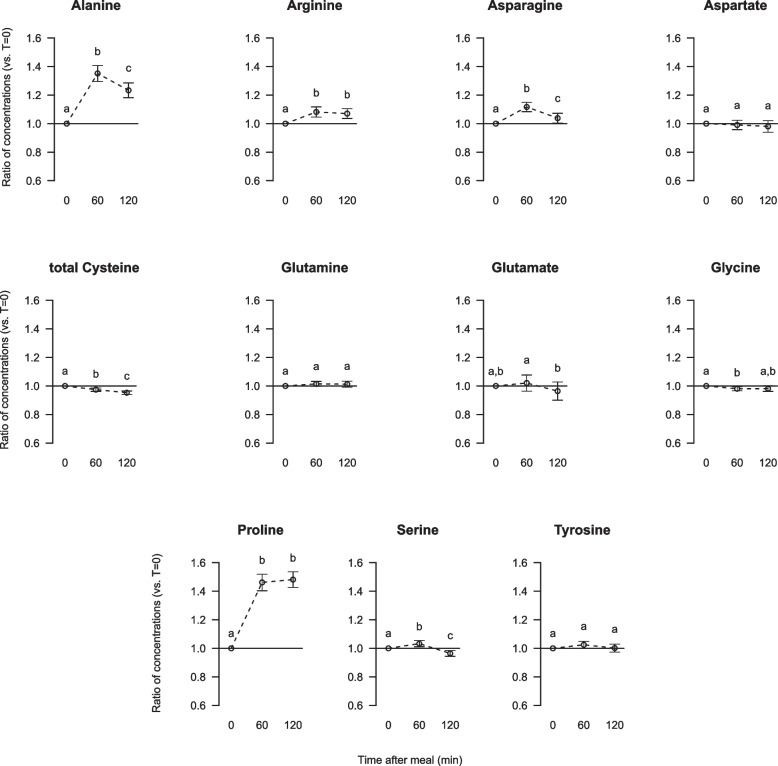
Relative changes from fasting to postprandial serum concentrations of non-essential amino acids. Data are presented as ratios with 5, 95% CI for 63 participants. Different letters (**a**, **b**, **c**) indicate significant differences between the time points 0 (fasting), 60 and 120 min; *P* < 0.05 was considered significant

The most pronounced changes in amino acid concentrations from fasting to 60 min after consumption of the standardised breakfast were seen for the non-essential amino acids (NEAA) alanine and proline (34% and 45% increase, respectively, Fig. 2). Whereas proline concentration maintained at this high concentration after 120 min, the alanine concentration was reduced to a level between fasting and 60 min concentrations. The serum concentrations of the other NEAA were less affected by breakfast intake (< 10% change); these were significantly increased (arginine and asparagine), significantly decreased (total cysteine and glycine), initially significantly increased followed by a significant decrease (serine), or were not changed (aspartate, glutamate, glutamine and tyrosine).

The fasting serum concentrations of isoleucine, leucine, methionine, tryptophan, valine, glutamate, and tyrosine were significantly higher in men compared to women (Table 4) . For the other measured amino acids, the fasting serum concentrations were similar between the genders.

**Table 4:** Fasting serum concentrations of amino acids

Amino acids (µmol/l)	Total (*N* = 63)	Men (*N* = 27)	Women (*N* = 36)	*P* sex
Essential amino acids				
Histidine	80.7 (78.6, 82.8)	79.4 (76.3, 82.7)	81.7 (78.8, 84.6)	0.55
Isoleucine	68.4 (64.9, 72.0)	78.6 (73.4, 84.3)	61.1 (58.1, 64.3)	1.0 × 10^–5^
Leucine	136 (130, 142)	155 (146, 164)	122 (118, 128)	1.2 × 10^–6^
Lysine	196 (189, 202)	200 (190, 210)	193 (184, 202)	0.60
Methionine	28.0 (27.0, 29.1)	30.0 (28.4, 31.6)	26.6 (25.4, 27.8)	6.0 × 10^–3^
Phenylalanine	72.6 (70.6, 74.6)	74.6 (71.5, 77.7)	71.0 (68.5, 73.6)	0.22
Threonine	140 (133, 147)	139 (128, 151)	141 (132, 151)	0.77
Tryptophan	71.1 (68.8, 73.5)	76.4 (72.9, 80.0)	67.2 (64.6, 69.9)	1.7 × 10^–3^
Valine	270 (259, 282)	301 (284, 320)	248 (238, 259)	2.8 × 10^–5^
Non-essential amino acids				
Alanine	381 (364, 400)	394 (368, 422)	372 (348, 397)	0.39
Arginine	97.0 (91.9, 102)	94.6 (86.2, 103.7)	98.9 (92.4, 106.0)	0.60
Asparagine	53.0 (51.1, 54.9)	51.6 (49.1, 54.3)	54.1 (51.3, 56.9)	0.33
Aspartate	21.2 (20.1, 22.4)	21.1 (19.5, 22.9)	21.2 (19.6, 23.0)	0.96
Total cysteine	299 (292, 306)	301 (293, 311)	296 (286, 307)	0.63
Glutamate	67.9 (62.5, 73.7)	76.8 (69.1, 85.3)	61.5 (54.7, 69.1)	0.035
Glutamine	554 (539, 569)	558 (540, 576)	550 (528, 574)	0.84
Glycine	249 (236, 263)	234 (223, 246)	262 (240, 286)	0.14
Proline	167 (155, 178)	174 (155, 194)	161 (147, 176)	0.52
Serine	133 (128, 138)	128 (121, 136)	137 (130, 144)	0.22
Tyrosine	70.1 (66.6, 73.8)	76.2 (71.8, 81.0)	65.6 (61.0, 70.5)	0.022

Data are presented as geometric means with 5, 95% CI. Men and women were compared using independent samples T test. *P* < 0.05 was considered significant

**Fig. 3:**
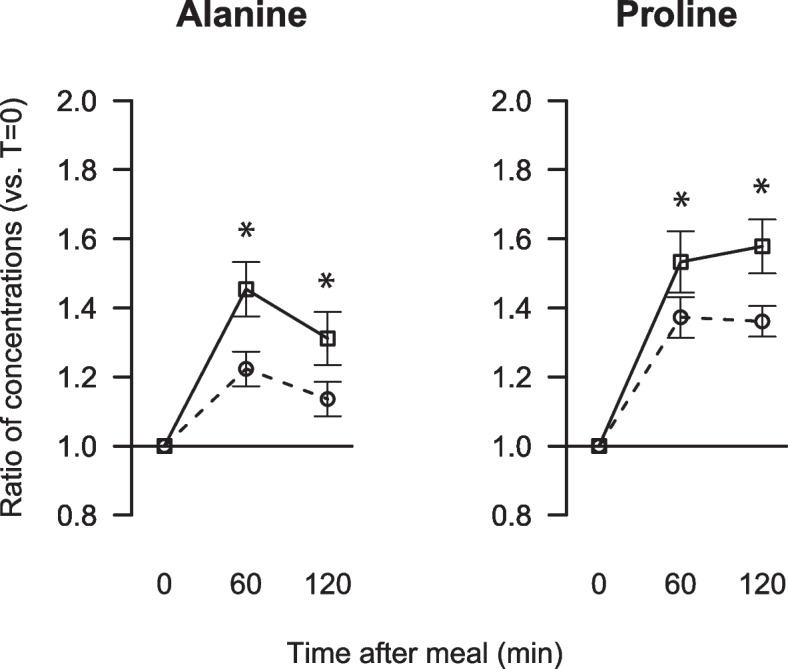
Relative changes from fasting to postprandial serum concentrations of alanine and proline in men (dotted line with circles) and women (solid line with squares). Data are presented as ratios with 5, 95% CI for 27 men and 36 women. The asterisks indicate significant differences between men and women; *p* < 0.05 was considered significant

The serum concentrations of several amino acids were differently affected by breakfast intake between the genders. The relative increases in serum concentrations from fasting to 60 min and to 120 min were considerably larger in women compared to men for alanine and proline (Fig. 3). For glycine, methionine, glutamine, threonine and phenylalanine, the ratios of postprandial to fasting serum concentrations were marginally but statistically significantly higher in women compared to men at either 60 or 120 min post breakfast, whereas both ratios for the other 13 amino acids were similar between the genders (data not presented).

### Effects of breakfast intake on the kynurenine pathway metabolites

**Fig. 4:**
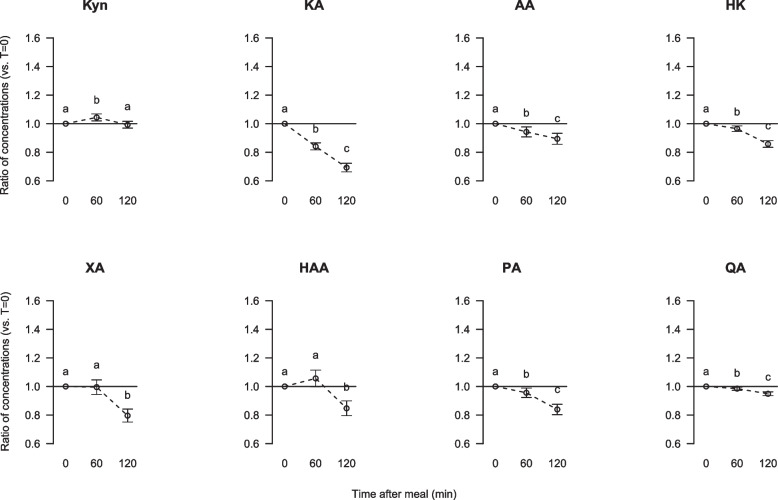
Relative changes from fasting to postprandial serum concentrations of the metabolites in the kynurenine pathway Kyn, kynurenine; KA, kynurenic acid; AA, anthranilic acid; HK, 3-hydroxykynurenine; XA, xanthurenic acid; HAA, 3-hydroxyanthranilic acid; PA, picolinic acid; and QA, quinolinic acid. Data are presented as ratios with 5, 95% CI for 63 participants. Different letters (**a**, **b**, **c**) indicate significant differences between the time points 0 (fasting), 60 and 120 min; *P* < 0.05 was considered significant

The serum concentrations of all measured kynurenine metabolites (kynurenic acid, anthranilic acid, 3-hydroxykynurenine, xanthurenic acid, 3-hydroxyanthranilic acid, picolinic acid and quinolinic acid) were significantly decreased 120 min after breakfast intake when compared to fasting concentrations when samples from men and women were assessed together (Fig. 4), whereas tryptophan (Fig. 1) and kynurenine (Fig. 4) concentrations were not affected postprandially. Significant reductions were seen already at 60 min after breakfast for serum concentrations kynurenic acid, anthranilic acid, 3-hydroxykynurenine, picolinic acid and quinolinic acid.

The fasting serum concentrations of tryptophan (Table 4) and the tryptophan metabolites kynurenine, kynurenic acid, xanthurenic acid, 3-hydroxyanthranilic acid and picolinic acid (Table 5) were significantly higher in men compared to women. For anthranilic acid, 3-hydroxykynurenine and quinolinic acid, the fasting serum concentrations were similar between the genders. The decrease in serum concentrations of tryptophan and all measured kynurenine pathway metabolites were similar between men and women after both 60 and 120 min (data not presented).

**Table 5:** Fasting serum concentrations of metabolites in the kynurenine pathway

	Total (*N* = 63)	Men (*N* = 27)	Women (*N* = 36)	*P* sex
Kynurenine (µmol/l)	1.54 (1.46, 1.62)	1.66 (1.55, 1.78)	1.44 (1.35, 1.55)	0.032
Kynurenic acid (nmol/l)	57.7 (52.9, 63.0)	67.7 (59.8, 76.7)	50.8 (45.5, 56.7)	9.0 × 10^–3^
Anthranilic acid (nmol/l)	16.2 (14.8, 17.6)	17.8 (15.4, 20.6)	15.0 (13.5, 16.6)	0.21
3-Hydroxykynurenine (nmol/l)	58.4 (54.5, 62.5)	62.4 (56.3, 69.1)	55.4 (50.5, 60.8)	0.30
Xanthurenic acid (nmol/l)	18.3 (16.6, 20.2)	22.0 (19.4, 24.9)	15.8 (13.9, 18.0)	7.4 × 10^–3^
3-Hydroxyanthranilic acid (nmol/l)	46.4 (42.5, 50.7)	54.0 (46.5, 62.8)	41.2 (37.6, 45.3)	0.025
Picolinic acid (nmol/l)	38.2 (35.2, 41.5)	45.9 (40.6, 51.8)	33.0 (30.2, 36.1)	8.5 × 10^–4^
Quinolinic acid (nmol/l)	412 (388, 437)	415 (376, 458)	410 (379, 443)	0.96

Data are presented as geometric means with 5, 95% CI. Men and women were compared using independent samples T test. *P* < 0.05 was considered significant

### Effects of breakfast intake on serum glucose, insulin, and metabolites related to glucose regulation

**Fig. 5:**
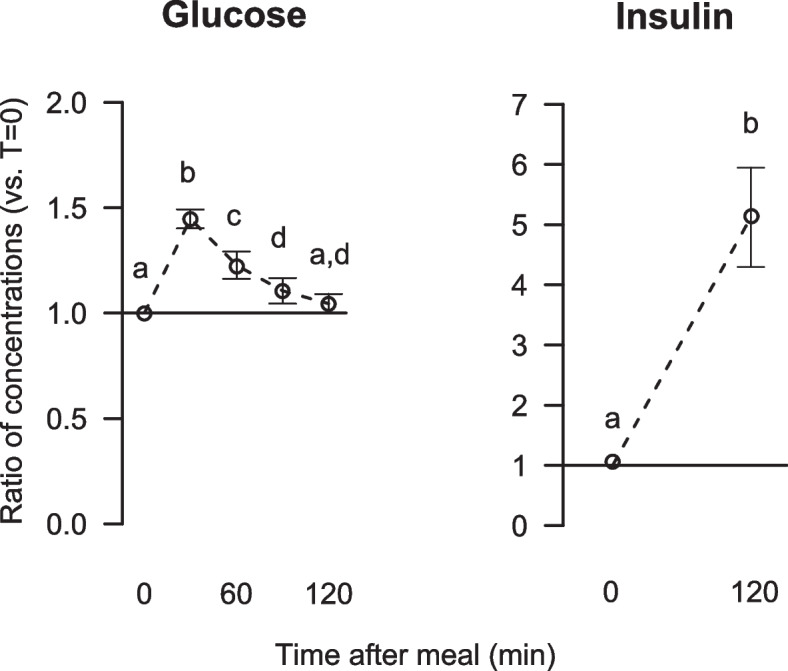
Relative changes from fasting to postprandial serum concentrations of glucose and insulin. Data are presented as ratios with 5, 95% CI for 63 participants. Different letters (**a**, **b**, **c**, **d**) indicate significant differences between the time points 0 (fasting), 60 and 120 min; *P* < 0.05 was considered significant

To confirm the participants’ change from the catabolic to the anabolic state, glucose and insulin were measured in fasting serum and at set times for 120 min following the meal. After intake of breakfast (containing 80 g carbohydrates), the serum glucose concentration peaked after 30 min, showing a significant 44% increase from fasting concentration (Fig. 5). After 120 min, the serum glucose concentration was almost back to fasting concentration with geometric mean concentration 3% above that of fasting glucose, whereas the geometric mean concentration of insulin was significantly increased five-fold (Fig. 5). After intake of the standardised breakfast, the relative increases in serum glucose and insulin concentrations were similar in men and women (data not presented).

**Fig. 6:**
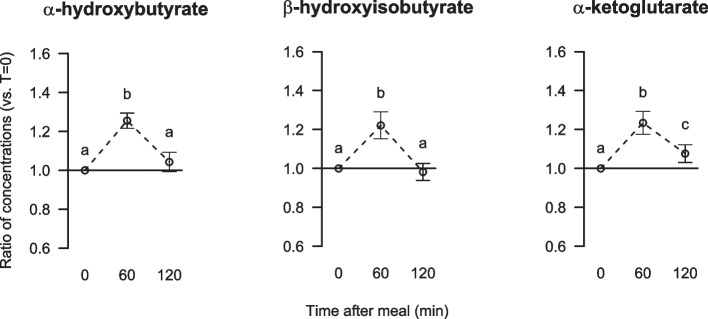
Relative changes from fasting to postprandial serum concentrations of α-hydroxybutyrate, β-hydroxyisobutyrate and α-ketoglutarate. Data are presented as ratios with 5, 95% CI for 63 participants. Different letters (**a**, **b**, **c**) indicate significant differences between the time points 0 (fasting), 60 and 120 min; *P* < 0.05 was considered significant

The serum concentrations of α-hydroxybutyrate and β-hydroxyisobutyrate were significantly increased by 25% and 20%, respectively, after 60 min, and returned to fasting concentrations after 120 min (Fig. 6). The α-ketoglutarate concentration was significantly increased by 22% after 60 min, and after 120 min the concentration was significantly reduced but was still higher than the fasting concentration (Fig. 6). The fasting serum concentrations were similar between the genders for α-hydroxybutyrate and α-ketoglutarate, whereas the concentration of β-hydroxyisobutyrate was significantly higher in men compared to women (Table 6). The changes in α-hydroxybutyrate, β-hydroxyisobutyrate, α-ketoglutarate from fasting to 60 and 120 min were similar between the genders (data not presented).

**Table 6:** Fasting serum concentrations of α-hydroxybutyrate, β-Hydroxyisobutyrate and α-ketoglutarate

	Total (*N* = 63)	Men (*N* = 27)	Women (*N* = 36)	*P* sex
α-Hydroxybutyrate (µmol/l)	47.4 (43.9, 51.2)	52.3 (46.7, 58.6)	43.8 (39.7, 48.5)	0.081
β-Hydroxyisobutyrate (µmol/l)	19.6 (18.6, 20.7)	21.4 (19.6, 23.4)	18.3 (17.2, 19.5)	0.026
α-Ketoglutarate (µmol/l)	8.71 (8.32, 9.13)	9.18 (8.62, 9.79)	8.35 (7.83, 8.91)	0.16

Data are presented as geometric means with 5, 95% CI. Men and women were compared using independent samples T test. *P* < 0.05 was considered significant

## Discussion

In the present study we demonstrate the importance of obtaining information concerning the prandial status of subjects who provide blood samples for epidemiological and intervention studies which evaluate circulating amino acids, kynurenine pathway metabolites and the amino acid catabolites α-hydroxybutyrate, β-hydroxyisobutyrate and α-ketoglutarate. We also present findings suggesting that for some amino acids, the postprandial amino acid metabolism differ for men and women.

The fasting serum amino acid profile of participants in the present study was characterised by the highest concentrations of glutamine and alanine, both of which are known to be synthesised and secreted from muscle during fasting [2]. As expected, the postprandial changes in serum amino acid concentrations did not reflect the amino acid composition of the breakfast, since a majority of the dietary amino acids are metabolised in the enterocytes and do not enter the portal circulation [27]. The breakfast contained relatively high amounts of glutamine and glutamate, and these amino acids are mainly metabolised by the small intestine in the first pass to produce large amounts of alanine, citrulline and proline [27]. In line with this, we observed no postprandial increase in glutamine and glutamate, whereas both alanine and proline increased significantly following the light breakfast. In addition, the increased insulin concentration following a meal inhibits hepatic gluconeogenesis by preventing the extraction of alanine and other glucogenic substrates from circulation but does not inhibit the release of alanine from muscle [1], causing a transitory increase in the circulating alanine concentration. Hence, the marked increases in postprandial alanine and proline concentrations are likely a result of a combination of these factors.

In the fasting state, the BCAAs leucine, isoleucine and valine in muscle are catabolised through transamination to form glutamate, which together with pyruvate efficiently forms alanine and α-ketoglutarate in a second transamination reaction. Elevated fasting concentrations of α-hydroxybutyrate (from threonine and methionine), β-hydroxyisobutyrate (catabolite of valine) and α-ketoglutarate (produced from glutamate by alanine aminotransferase and aspartate aminotransferase) have been linked to impaired glucose tolerance and insulin resistance [9–11]. Little is known, however, about non-fasting concentrations of α-hydroxybutyrate, β-hydroxyisobutyrate and α-ketoglutarate. The breakfast contained moderate amounts of BCAAs; but, since most of the enterally delivered BCAAs are extracted by the splanchnic bed and their nitrogen are mainly used for alanine synthesis, only limited amounts of dietary BCAAs enter portal circulation [27], and the postprandial serum concentrations of the three BCAAs were reduced from 60 to 120 min. We observed an increase in α-hydroxybutyrate, β-hydroxyisobutyrate and α-ketoglutarate in serum collected 60 min after the light breakfast, likely reflecting increased postprandial catabolism of amino acids such as glutamate and valine in the present study. These findings indicate that elevated concentrations of these candidate biomarkers of insulin sensitivity should be interpreted with caution if the prandial status of the subject is not known.

The kynurenine pathway is quantitatively the most important route for tryptophan catabolism [13], and studies in rats indicate that the first step in the kynurenine pathway is inhibited by glucose [15]. Consequently, the increased glucose concentration after breakfast intake may contribute to reduced catabolism of tryptophan through this pathway. In line with this, we observed reductions in postprandial serum concentrations in all downstream metabolites of kynurenine. The tryptophan content in the breakfast was below the detection limit, and the serum tryptophan concentration was not increased after breakfast intake despite the apparent downregulation of the kynurenine pathway, which is not surprising since the tryptophan concentration is roughly 1000-fold higher than most kynurenines in serum. These observations emphasise the importance of knowledge of fasting status when interpreting the circulating concentrations of kynurenines.

Following an overnight fast, our male and female participants had comparable serum concentrations of glucose, insulin, and the amino acid catabolites α-hydroxybutyrate and α-ketoglutarate, and the postprandial increases in concentrations of glucose and insulin were similar between the genders. However, our male participants had higher fasting concentrations of isoleucine, leucine, methionine, tryptophan, valine, glutamate and tyrosine when compared to the female participants. The men in the present study also had higher fasting serum concentration of β-hydroxyisobutyrate when compared to female participants, which may indicate that insulin sensitivity was poorer in men in our study since β-hydroxyisobutyrate has been shown to be a biomarker of impaired glucose intolerance and marker of future risk for type 2 diabetes [11, 28]. Hence, relative to the women in our study, our male participants had an unfavourable fasting amino acid profile that has been associated with insulin resistance [5, 29, 30]. Also, the less pronounced postprandial increases in alanine and proline in men could point towards inferior insulin sensitivity when compared to women. Similar gender differences in amino acid compositions have been observed by others [29] in a population with comparable BMI and slightly higher mean age compared to the present study. In addition to higher tryptophan serum concentration, the men also had higher fasting concentrations of kynurenine, kynurenic acid, xanthurenic acid, 3-hydroxyanthranilic acid and picolinic acid when compared to women. This is partly in agreement with observations of higher concentrations of kynurenine pathway metabolites in men in a young, healthy population with normal BMI [31], in healthy Caucasians age 18–40 years [32], and in the Hordaland Health Study with age ranges 46–47 and 70–72 years [33].

The present study has some strengths and limitations. Strengths include that all participants consumed a well-characterized light meal as breakfast following an over-night fast, and a relatively large sample size that consisted of both men and women. Serum samples were analysed using established methods with high precision and we strictly followed a defined protocol for pre-analytical handling of samples, and samples were thawed for the first time for these analyses and showed no signs of degradation. Limitations include the generalisability of the findings for other populations such as those with normal bodyweight, other age groups, other ethnicities, and patients with established metabolic disturbances or diseases, and the use of meals with other ingredients and serving sizes. It would also be of interest to see how the biomarkers are affected after 2 h postprandially.

To conclude, our findings demonstrate that a light breakfast with low protein content (14 g) was sufficient to induce changes in circulating concentrations of several amino acids and related metabolites, and these changes did not reflect the amino acid composition of the meal. We also present findings of some sex-differences for both fasting and postprandial biomarker concentrations, which may impact interpretations of biomarker related health and disease risk and should be further investigated. Our findings underline the importance of having information regarding the prandial state of the study participants or patients in epidemiological and intervention studies when exploring biomarker data related to amino acids and their metabolites.

## Data Availability

The datasets used and analysed during the current study are available from the corresponding author on reasonable request.
